# Catch me if you can: Leukemia Escape after CD19-Directed T Cell Immunotherapies

**DOI:** 10.1016/j.csbj.2016.09.003

**Published:** 2016-09-28

**Authors:** Marco Ruella, Marcela V. Maus

**Affiliations:** a Center for Cellular Immunotherapies, Perelman School of Medicine at the University of Pennsylvania, Philadelphia, PA, United States; b Department of Pathology and Laboratory Medicine, Perelman School of Medicine at the University of Pennsylvania, Philadelphia, PA, United States; c Abramson Cancer Center, University of Pennsylvania, Philadelphia, PA, United States; d Massachusetts General Hospital, Harvard Medical School, Boston, MA, United States

**Keywords:** Chimeric antigen receptor T cells, Blinatumomab, Bispecific antibodies, Adoptive cell therapy, dualCART, tanCAR, biCAR, CART19, CTL019, Leukemia

## Abstract

Immunotherapy is the revolution in cancer treatment of this last decade. Among multiple approaches able to harness the power of the immune system against cancer, T cell based immunotherapies represent one of the most successful examples. In particular, biotechnological engineering of protein structures, like the T cell receptor or the immunoglobulins, allowed the generation of synthetic peptides like chimeric antigen receptors and bispecific antibodies that are able to redirect non-tumor specific T cells to recognize and kill leukemic cells. The anti-CD19/CD3 bispecific antibody blinatumomab and anti-CD19 chimeric antigen receptor T cells (CART19) have produced deep responses in patients with relapsed and refractory B-cell acute leukemias. However, although the majority of these patients responds to anti-CD19 immunotherapy, a subset of them still relapses. Interestingly, a novel family of leukemia escape mechanisms has been described, all characterized by the apparent loss of CD19 on the surface of leukemic blasts. This extraordinary finding demonstrates the potent selective pressure of CART19/blinatumomab that drives extreme and specific escape strategies by leukemic blasts. Patients with CD19-negative relapsed leukemia have very poor prognosis and novel approaches to treat and ideally prevent antigen-loss are direly needed. In this review we discuss the incidence, mechanisms and therapeutic approaches for CD19-negative leukemia relapses occuring after CD19-directed T cell immunotherapies and present our future perspective.

## 1 Background

A novel era for cancer treatment has started: redirecting the immune system to recognize cancer cells has led to unprecedented responses in many tumor subtypes [1]. The biotechnological engineering of known protein structures (e.g. T cell receptor or immunoglobulins) led to the generation of synthetic peptides like chimeric antigen receptors and bispecific antibodies that are able to redirect non-tumor specific T cells to recognize and kill leukemic cells. In the setting of B cell leukemias and lymphomas, anti-CD19 chimeric antigen receptor T cells (CART19) and anti-CD19/CD3 bispecific antibody (blinatumomab) led to unprecedented results in multiple clinical trials [2], [3]. These results led to the FDA-approval of blinatumomab in 2014 [4] and to the assignment of the status of "breakthrough therapy" for several CART19 products in 2015.

Blinatumomab is a bispecific T cell engager (BiTE), that includes two different single-chain variable fragments (scFv) derived from monoclonal antibodies against CD19 and CD3ε. This small artificial protein structure is able to engage patient's own T cells and bring them in close proximity to cancer cells leading to potent activation and anti-tumor response [5]. One of the key factors to ensure the high efficiency of this peptide is the structural design where the anti-CD19 scFv has a higher affinity compared to the anti-CD3 so that a T cell can recognize and kill multiple B cells that are bound to BiTE molecules [6], [7], [8]. With blinatumomab T cells are temporarily redirected against cancer cells and continuous infusion of this drug is required to ensure activity [9]. The BiTE concept was originally developed in Germany about 15 years ago [6] and is now extensively used in the clinic for B-cell acute lymphoblastic leukemia (B-ALL) [7], [10]. On the other hand, CART are patient's own T cells that are reengineered ex vivo to express a chimeric antigen receptor (CAR) that empowers them to recognize one antigen expressed on the surface of leukemic cells. In the clinical setting, CART are activated, genetically modified, expanded and then reinfused back to the patient in order to reconstitute a permanent anti-tumor immune system. The concept of CAR started in the late ‘80 when groups in Israel and Japan [11], [12] conceptualized the design of a synthetic immune receptor generated by the fusion of a scFv from a monoclonal antibody with the intracellular signaling domain of the T cell receptor (TCR, specifically the CD3ζ chain). One of the reasons of the success of CART19 is the use of 2nd generation CAR structures that, together with the CD3ζ chain, also include a costimulatory domain, i.e. CD28 or 4-1BB (as opposed to 1st generation CAR that included only the CD3ζ signaling domain without costimulation domain). This way both signal 1 (antigen recognition) plus signal 2 (costimulation) are summarized in a single construct, recapitulating full T cell activation [13]. These two potent anti-CD19 immunotherapies enable a non-tumor specific T cell to recognize antigens expressed on the surface of the tumor cell with the affinity of a monoclonal antibody and triggering T cell activation like a TCR.

It took many years for these concepts to emerge as an effective and successful clinical therapy, but in the last 5–7 years blinatumomab and CART19 have proven their immense potential leading to deep tumor response in patients with otherwise extremely poor prognosis [14], [15], [16], [17]. In an early clinic trial, blinatumomab led to the clearance of minimal residual disease (MRD) in 80% of adult B-ALL patients treated [18], [19]. A confirmatory study enrolled 116 adult B-ALL patients with MRD (~ 60% were in first morphologic complete remission, CR) and showed 78% MRD response with improved relapse-free survival (RFS) and overall survival (OS) [20], [21]. In the setting of adult relapsed or refractory (r/r) B-ALL patients with overt disease (> 5% blasts in the bone marrow, BM) blinatumomab led to ~ 69% CR (and CR with partial hematological recovery, CRi) with 88% being MRD-negative. About half (13/25) of the patients reaching CR/CRi received an allo-SCT [22]. In a confirmatory phase II trial, 189 adult patients with r/r B-ALL were treated with blinatumomab: 43% of patients reached CR/CRi. Median RFS and OS were 6.1 and 5.9 months respectively. Only 43/189 patients continued treatment beyond cycle 2 and, again, about half of CR patients received allo-SCT. Patients with higher tumor burden (> 50% blasts in the BM) had the worse outcome (29% vs. 73% CR rate) [10]. Other studies showed promising activity of blinatumomab in pediatric ALL [23] and in Philadelphia-positive ALL [24]. Regarding CART19, multiple groups have shown that this approach can induce complete responses in 60–90% of B-ALL patients in clinical trials, both in pediatrics and adults [14], [15], [25]. In a recent update of the pediatric study of the Univ. of Pennsylvania CART19 (CTL019), out of 59 patients 55 (93%) were in CR at 1 month. With a median follow-up of 12 months RFS and OS were 55% and 79% respectively [26].

Despite the excellent clinical responses of r/r B-ALL patients to CD19-directed T cell therapies, a significant number of patients still relapses. In the blinatumomab study, 8/12 patients who did not receive subsequent transplantation relapsed, and 2/13 of the ones receiving allo-SCT relapsed [22]. For CART19 of 93% CR 1 month after therapy only 55% of the patients are disease-free at 1 year, indicating that about half of the patients relapse. Of note, no relapses have been observed after 1 year [26]. Therefore despite very high short-term response rates, a significant proportion of patients still relapses, especially in the blinatumomab cohorts where no prolonged T cell memory is established. In this context, two main types of relapses are recognized: i. CD19 + relapses — where the leukemia phenotype is the same as before treatment: typically these relapses are linked to poor T cell function or early CAR T cell disappearance; ii. CD19-negative relapses — where the disease recurs with apparent loss of CD19: these relapses can occur despite a strong activity of CART19 or blinatumomab and represent a novel mechanism of tumor escape. In this review we will focus on this second type of relapses, discussing the incidence, the current prognosis, and possible management strategies for CD19-negative relapses.

## 2 Tumor Escape after CD19-Targeted Therapies

Since targeted therapies have been introduced for the treatment of cancer, novel specific mechanisms of tumor escape have been observed. As an example, the use of tyrosine kinase inhibitors for chronic myeloid leukemia can lead to mutations in the BCR-ABL protein (T315I), which confer resistance to the drug [27]; in melanoma, the efficacy of BRAF inhibitor can be abolished by mutations in BRAF or other alternative pathways [28]. The apparent CD19-loss is a specific tumor escape mechanism observed for the first time in the setting of potent CD19-directed immunotherapies. These findings indicate how cancer is a heterogeneous disease and can evolve over time adapting to the environment.

In the setting of blinatumomab, when 16 of 20 adult B-ALL patients with MRD + disease achieved CR, 6 relapses occurred (all after completing drug treatment), of which 2 were CD19-negative and 4 were CD19 +. Four clinical relapses (2 CD19 +, 2 CD19-negative) were observed in patients who did not undergo transplantation after completion of blinatumomab treatment and 2 in patients receiving transplantation (all CD19 +) [19], [18]. In a more recent study in active adult r/r B-ALL in CR after blinatumomab 3/10 relapses were CD19-negative [22], [29]. A recent report in abstract form described the occurrence of a CD19-negative B-ALL relapse in a 69-year old patient treated with blinatumomab; the authors suggest the use of a different flow cytometry gating strategy that does not rely exclusively on CD19 for early detection of these antigen-loss relapses [30].

In the setting of CART19, in a study run at the Children's Hospital of Philadelphia and at the Univ. of Pennsylvania, 59 pediatric patients with r/r B-ALL were treated with 2nd generation, 4-1BB costimulated CART19 cells. In this cohort of heavily pretreated patients 93% reached CR with 88% MRD negativity. At a median follow-up of 1 year, 34 patients had ongoing CR with only 6 receiving subsequent therapy. Twenty patients subsequently relapsed: 13 with CD19-disease [14], [31], [26]. In a study using CD28-costimulated CART19, two patients who achieved MRD-negative CR and were judged ineligible for allo-SCT both relapsed with CD19-negative leukemia at 3 and 5 months. On the contrary, ten patients who underwent allo-SCT in a CAR-induced MRD-negative state remained disease-free with no unexpected peritransplant toxicities [32]. More recently, the Seattle group reported that 9 patients out of 29 (31%) adult B-ALL patients relapsed, and 3 (33%) of them were CD19-negative (1 myeloid phenotype switch) [17]. The group at Memorial Sloan Kettering Cancer Center reported the occurrence of CD19-negative relapses in 14% of adult B-ALL patients treated with CART19 (CD28 costimulation). In this trial, CR rate was 87% with 81% MRD negativity [16], [33]. Therefore CD19-negative relapses are observed in B-ALL patients treated with different CART19 products, independently of the construct (different costimulation domains) and expansion/clinical protocol. However, we might speculate that long-term persistence of CAR T cells and the absence of subsequent allo-SCT could increase the chances of antigen-loss relapse. No clear risk factor for the development of CD19-negative relapses has been recognized, however, in our experience the previous exposure to blinatumomab pre-CART19 could possibly represent a risk factor.

## 3 Possible Mechanisms of Escape

Due to the novelty of CD19 loss as an escape mechanism, there are only few studies that investigated this condition and its possible mechanism. A paper published by our group, led by Dr. Elena Sotillo at the Children Hospital of Philadelphia, demonstrated that, at least in a subset of CD19-negative leukemias emerging after CD19-directed therapy, mutations affecting the CD19 gene and CD19 splicing variants lacking the CAR-recognized epitope are strongly enriched compared to samples before CART19 treatment. In particular, exon 2 of CD19 was frequently spliced out, leading to the disappearance of the CD19 epitope that is recognized by the FMC63-based antigen-binding moiety of CART19. This study suggests indeed the possibility that in some patients the CD19 protein is still present but it is truncated, lacking the epitope that is necessary to trigger CART19 recognition for lysis and CD19 detection by flow cytometry [34].

A variant of the CD19-negative relapses is myeloid lineage switch. This phenomenon has been observed in murine models where mice bearing E2a:PBX1 leukemia and treated with murine CART19 developed at long-term myeloid CD19-negative relapses (as opposed to CD19-negative lymphoid relapse at short-term) [35]. In humans, the Seattle group reported the occurrence of myeloid switch relapse after CART19 in 2 out of 7 patients with B-ALL harboring rearrangement of the mixed lineage leukemia (MLL) gene. The relapses were demonstrated to be clonally related to the pre-treatment disease. Interestingly different mechanisms seem to have led to the myeloid relapses: for the first patient, retention of the immunoglobulin (Ig) rearrangement was observed in relapsed myeloid blasts suggesting reprogramming or de-differentiation of a previously committed B lymphoid blast; for the second patient absence of the Ig rearrangement could indicate myeloid differentiation of a non-committed precursor or, most likely, the selection of a preexisting CD19-negative myeloid clone by CART19 treatment [36]. Of note, a similar phenomenon was observed in a 3-month-old B-ALL infant with MLL-AFF1 rearrangement that was treated with blinatumomab and relapsed on day 15 after treatment initiation with a monoblastic AML (with identical karyotype as pre-blinatumomab) [37]. Lineage switch is a rare event in ALL and CD19-specific selective pressure may lead to a different blast differentiation program or to the selection of minor myeloid CD19-negative leukemic subpopulations giving rise to overt myeloid relapses; however, the exact mechanisms are still poorly understood. Our group recently suggested that minor CD19-negative subpopulations, already observed in our initial report in 2013 [38], can pre-exist in the leukemia bulk and can be selected for under the strong pressure of CART19. In this study, we analyzed 6 B-ALL samples that carried various rearrangements identifiable by fluorescent in situ hybridization (FISH) and in all of them we could identify a minor CD19-negative subset that harbored the same FISH profile as the bulk leukemia. Further studies to investigate the role of these minor subpopulations in CART19-treated patients are ongoing [39]. Lastly, another study described a patient with chronic lymphocytic leukemia (CLL) (in the BM) and Richter syndrome (in the lymph nodes) who was treated with CART19 and achieved 50% nodal response and 80% reduction of CLL in the BM. Approximately 6-month after CART19 the patient relapsed with a clonally related CD19-negative plasmablastic lymphoma (PBL) and a minor population of residual CD19-negative CLL. Clonal relationship between the pre-treatment CLL and both the subsequent CD19-negative PBL and CLL was confirmed by molecular analysis (immunoglobulin rearrangements). This report indicates that not only early-precursor B-ALL but also differentiated B-cell neoplasms (e.g. CLL/Richter syndrome) can develop antigen-loss escape [40].

Therefore several possible mechanisms have been described to explain CD19-loss escapes but many more will be likely defined in the next few years as more and more patients are being treated with blinatumomab and CART19 in the clinic. Regardless of the mechanisms of CD19-negative relapse, these patients have a very poor prognosis and novel therapeutic strategies are urgently needed.

## 4 Strategies to Avoid Antigen-Loss Relapses

Antigen-loss relapses are an emerging limitation of potent CD19-targeted immunotherapies and, as discussed, are caused by the use of very active selection agents that can recognize only one antigen on leukemia cells. Novel strategies to avoid antigen loss are being tested and include allogeneic transplantation and the co-targeting of multiple markers on leukemic cells.

Allogeneic stem cell transplantation (allo-SCT) represents a possible additional treatment that could potentially reduce the risk of antigen-loss relapse after CR is obtained with CD19-directed immunotherapies. In allo-SCT, patient's own hematopoiesis is ablated via high-dose chemotherapy or radiation and new hematopoietic stem and progenitor cells from a compatible donor are reinfused with the goal of regenerating normal hematopoiesis. Together with the anti-leukemia effect of the conditioning regimen, a new immune system that can potentially recognize and destroy residual leukemic cells is established. Since the graft-anti-leukemia effect generated by allo-SCT is not specific for CD19 + cells, this approach could reduce relapse independently from CD19 expression. There is no randomized clinical trial that proves this hypothesis but the early phase clinical trials of CART19 and blinatumomab suggest that patients receiving allo-SCT after CD19-immunotherapies seem to have a lower frequency of developing CD19-negative relapses. However, patients could potentially develop CD19-negative relapses after CART19 even when followed by allo-SCT due to the persistence of CART19-selected CD19-negative leukemia initiating-cells. Moreover, many patients treated with blinatumomab or CART19 have already received allo-SCT or are not eligible for this treatment. Lastly, the toxicity related to allo-SCT is still high and this approach should be carefully evaluated in these heavily pretreated patients.

Another strategy to tackle CD19-negative relapses is to use agents targeting other markers on leukemic cells. Possible targets expressed in both normal B-cells and B-ALL blasts are CD22, CD123 and CD20. There are currently 3 clinical trials including CART22 for B-ALL. At the NCI 4/9 pediatric and young adult B-ALL patients (some relapsing with CD19-negative disease after CART19) treated with CART22 reached MRD-negative CR [41], [42], [43] At the University of Pennsylvania/Children's Hospital of Philadelphia two CART22 trials (NCT02588456; NCT02650414) recently opened for adults and pediatric patients with r/r B-ALL. Other agents like the anti-CD22 antibody conjugated to calicheamicin (inotuzumab ozogamicin) could potentially target CD19-negative relapse [44]. Another target that is expressed on CD19-negative blasts relapsing after CART19 is the IL-3 receptor α, CD123. Our group demonstrated that anti-CD123 CART (CART123) are able to eradicate CD19-negative blasts in preclinical xenograft models [39].

**Fig. 1:**
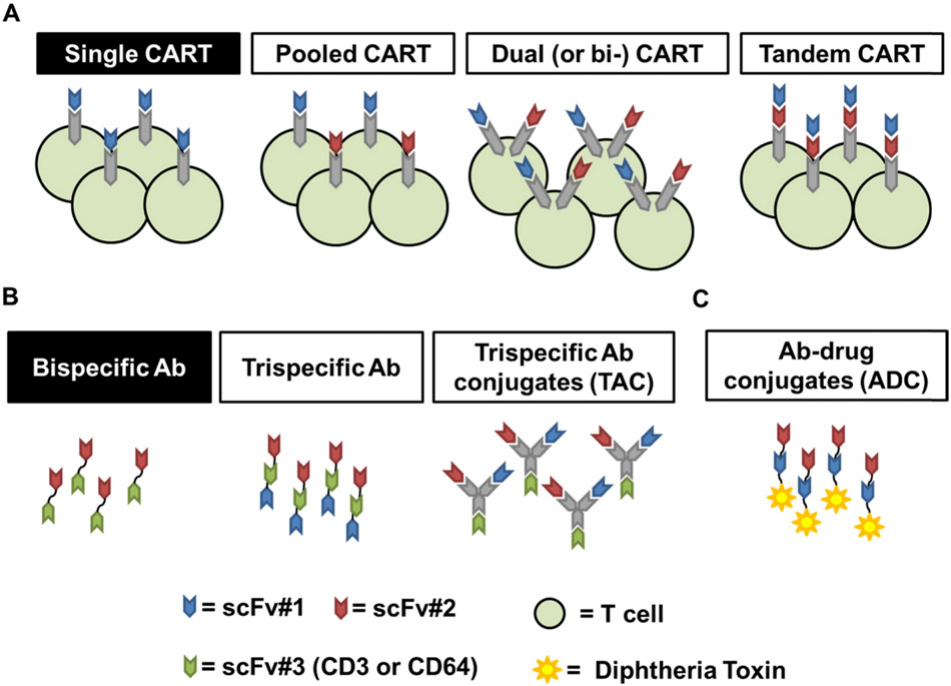
Different strategies to target 2 antigens on the surface of leukemic cells to avoid antigen-loss relapses. A. Chimeric antigen receptor T cells. Pooled CART are a 1:1 mixture of single–specificity CART: each cell remains able to recognize only one target (e.g. CD19 or CD123). Dual (or bi-) CART: every T cell bears 2 distinct CAR structures able to recognize 2 different targets (e.g. CD19 and CD123). Tandem CART: every T cell bears 1 CAR structure where 2 scFvs are built in series and are able to recognize 2 different targets (e.g. HER2 and IL13Rα2). B. Trispecific antibodies. Trispecific antibodies carry 3 scFvs, two recognize tumor targets (e.g. CD123 and CD33) and one engages the immune system (e.g. CD16). Trispecific antibody conjugates include 3 scFvs plus the respective Fabs; two recognize tumor targets (e.g. EGFR and HER2) and one engages the immune system (e.g.CD64). C. Bispecific antibody-toxin conjugates. Bispecific antibody-toxin conjugates carry 2 scFvs that recognize tumor targets (e.g. CD19 and CD22) linked to a toxin (e.g. Diphtheria toxin).

However, even though CD22, CD123 and in a lesser degree CD20 are expressed in B-ALL including CD19-negative relapses we might speculate that targeting a single-marker could lead inevitably to antigen-loss escape as observed for CD19. Therefore targeting more than one antigen on cancer cells represent a reasonable strategy to successfully avoid antigen-loss. This approach hasn't been tested clinically yet, but several pre-clinical studies have been published using both CART and bi- or tri- specific antibodies, as depicted in Fig. 1. The Baylor College of Medicine demonstrated that CART cells expressing both anti-HER2 CAR and anti-IL-13Rα2 zetakine (bi-CAR or dual CAR) can offset antigen escape in glioblastoma pre-clinical models. Moreover, in their model, biCART are also more active than the pooled combination of single-expressing anti-HER2 and anti-IL13Rα2 CAR T cells [45]. In a recent follow-up study they showed that a structural change to the biCAR where the two antigen-recognizing domains are put in series on top of the CAR backbone (Tandem CAR or tanCAR [46]) leads to better anti-tumor activity as compared to biCART [47]. In another report from the same group, pooled co-targeting of MUC1 and PSCA with CART in prostate cancer preclinical models led to reduced antigen escapes [48]. All these studies evaluated antigen-loss in preclinical models of solid tumors, however in clinical trials CART have not demonstrated to lead to overt antigen loss in solid tumors probably because of their limited activity recorded so far. Preliminary clinical data in glioblastoma patients treated with anti-EGFRvIII CART seem to suggest that residual tumor after therapy has lower expression of EGFRvIII [49]. Our group recently demonstrated that co-targeting CD19 and CD123 can treat and also prevent antigen loss in a clinically-relevant preclinical model of CD19-negative leukemia escape. We used leukemic blasts derived from a B-ALL patient before treatment with CART19 when the disease was CD19 + CD123 + and after CART19 when the patient relapsed with CD19- CD123 + leukemia. Using a model where immunodeficient mice were engrafted with a 1:1 mixture of the leukemic cells from this patient before and after CART19 treatment, we demonstrated that co-targeting with CART19/123 can prevent antigen-escape. We also demonstarted that, in primary B-ALL xenografts, dual CART are more potent than pooled CART [39]. Other important targets that are being evaluated for cotargeting strategies to prevent antigen-escape are CD22 [50] and CD20 [51]. In antibody therapeutics, targeting multiple antigens with tri-specific antibodies (2 tumor targets +/− an immune system-engaging domain like CD64) is under investigation for solid tumors (targeting HER proteins, EGFR and others) [52], [53]. Trispecific (CD123, CD33, CD16) antibodies capable of redirecting NK cells (through CD16) to kill leukemia have also been developed [54]. A single-chain triple-body with specificity for CD19 and CD33 was shown to mediate effective lysis of mixed lineage leukemia cells by dual-targeting and engagement of NK cells (via CD16) [55]. Anti CD22/CD19 immunotoxins are being evaluated in the clinic (NCT00450944, NCT00889408, NCT02370160) and in the future could be important modalities to prevent antigen-loss relapses [56], [57]. Lastly, mixed therapeutic combinations of CART19 (or blinatumomab) plus other anti-leukemia antibodies or antibody-drug conjugates (ADC) (such as the anti-CD20 rituximab or anti-CD22 inotuzumab ozogamicin) could be evaluated to avoid CD19-negative escape.

## 5 Summary and Outlook

CART and blinatumomab immunotherapies are leading to high response rates and complete remissions in relapsed and refractory B-cell leukemia patients who otherwise carry very poor prognosis. Unfortunately, a significant subset of patients still relapses and most of the relapses (especially for CART19 therapy) are characterized by the apparent loss of CD19. The mechanism of this escape is still under investigation, while some reports suggest that convergence of acquired mutations and alternative splicing of CD19 enables leukemic cells to resist CART19 immunotherapy. Other mechanisms like the selection of minor CD19-negative clones or the induction of a myeloid switch have been described. It is likely that additional mechanisms will be described in the near future as more patients are treated with these potent anti-CD19 immunotherapies. Interestingly, to our knowledge, the vast majority of CD19-negative relapses have been recorded in the setting of B-ALL and only one case has been reported in NHL (CLL). This phenomenon can be related to the fact that more B-ALL patients are treated with CART19/blinatumomab as compared to NHL, but also it can reflect the different biology of undifferentiated blasts (B-ALL) versus mature B cells (NHL).

As we have learned with chemotherapy, the leukemia treatment requires multiple agents used together to avoid relapse [58]. The same concept may hold true for targeted immunotherapies as well as for small molecule inhibitors. The use of single agents can lead to specific escape mechanisms and it is likely that the use of combined approaches will reduce these events. For CART19 and blinatumomab the treatment of CD19-negative relapses is now managed with additional chemotherapy (with generally poor results), anti-CD22 agents like inotuzumab ozogamicin, anti-CD22 CART or other clinical trials; in these cases, the goal is to achieve remission and, if possible, proceed to an allo-SCT. Novel approaches will include upfront treatment with dual-targeting agents, taking advantage of markers that are commonly expressed in B-ALL like CD22, CD123 and CD20. However, as we have learned with chemotherapy, if the mechanism of resistance is linked to tumor adaptation, even targeting 2 antigens may not be enough to achieve cure in all patients with all cancers. Specific cocktails of CART or antibodies able to target specific antigens tailored for each patient could further enhance the efficacy and frequency of durable cancer remissions. The clinical experience with pediatric leukemias and testicular neoplasm demonstrates the power of combinational approaches to treat cancer and we believe that similar success will be reached with combined immunotherapies.

## Authorship Contribution

M.R. and M.V.M. wrote, reviewed and accepted the contents of the article.

## Competing Interests

M.R. works under a research collaboration involving the University of Pennsylvania and the Novartis Institute of Biomedical Research, Inc. M.R. and M.V.M. are inventors of intellectual property licensed by the University of Pennsylvania to Novartis.
